# Segregation distortion: Utilizing simulated genotyping data to evaluate statistical methods

**DOI:** 10.1371/journal.pone.0228951

**Published:** 2020-02-19

**Authors:** Alexander Coulton, Alexandra M. Przewieslik-Allen, Amanda J. Burridge, Daniel S. Shaw, Keith J. Edwards, Gary L. A. Barker

**Affiliations:** School of Biological Sciences, University of Bristol, Life Sciences Building, Bristol, United Kingdom; Julius Kuhn-Institut, GERMANY

## Abstract

Segregation distortion is the phenomenon in which genotypes deviate from expected Mendelian ratios in the progeny of a cross between two varieties or species. There is not currently a widely used consensus for the appropriate statistical test, or more specifically the multiple testing correction procedure, used to detect segregation distortion for high-density single-nucleotide polymorphism (SNP) data. Here we examine the efficacy of various multiple testing procedures, including chi-square test with no correction for multiple testing, false-discovery rate correction and Bonferroni correction using an *in-silico* simulation of a biparental mapping population. We find that the false discovery rate correction best approximates the traditional p-value threshold of 0.05 for high-density marker data. We also utilize this simulation to test the effect of segregation distortion on the genetic mapping process, specifically on the formation of linkage groups during marker clustering. Only extreme segregation distortion was found to effect genetic mapping. In addition, we utilize replicate empirical mapping populations of wheat varieties Avalon and Cadenza to assess how often segregation distortion conforms to the same pattern between closely related wheat varieties.

## Introduction

Segregation distortion is the phenomenon in which alleles in the progeny of a cross between two varieties or species deviate from expected Mendelian ratios. In an F2 population originating from a biparental cross, the expected ratio of genotypes AA:AB:BB (progeny homozygous for the allele from the first parent, heterozygotes and progeny homozygous for the allele from the second parent) in absence of segregation distortion is 1:2:1. Segregation distortion is observed across a wide range of taxa, including animals such as *Drosophila* [1,2] and mice [3], as well as crop species, including cotton [4], maize [5,6], potato [7], chickpea [8], barley [9] and wheat [10–12]. The primary explanation of the cause of segregation distortion is a selection pressure operating against one of the parental alleles at some stage of the development cycle, whether at meiosis through meiotic drive [1], through male gamete competition [4], or at the level of the zygote. An example of this is the pollen killer gene in wheat [13], for which there is an allele that causes pollen cells to degenerate until inviable, leading to an overrepresentation of the non-deleterious allele.

Segregation distortion can be problematic for crop breeders, who wish to generate varieties with novel genotypic compositions that are better suited to meeting the various aims of modern agriculture, such as increased yields or improved resistance to biotic or abiotic stresses. Distorted segregation at a locus could skew most lines in a recombinant inbred line population (RIL) away from the desired genotype, requiring breeders to create larger numbers of lines to compensate. It would be useful if we could attribute regions of segregation distortion to causative loci in the genome, as this would allow breeders to plan for the occurrence ahead of time. One important obstacle to this goal is another potential cause of segregation distortion: sampling error. With small RIL population sizes, it is possible that a specific parental allele is, by chance, sampled more often than its alternative in the progeny, leading to a distorted ratio of segregation. Conflating this for distortion caused by a selective pressure would be problematic, as the same pattern of distortion in the progeny would not be repeated if the RIL population was recreated. Planning for this distortion in breeding programmes would therefore be counterproductive. How much of the segregation distortion typically observed in RIL populations is due to chance?

Distinguishing distortion caused by selection from that caused by chance is difficult, because both have the potential to produce similar patterns of segregation in the progeny of a cross. What we can assume though is that if the selection strength is high enough, the intensity of distortion produced would be unlikely to have occurred by chance. This is complicated by the fact that the effects of chance change with population size, being more prevalent when population size is small, and eventually evening out as population size becomes larger. Our sole detection criterion for separating selection from chance as the cause of segregation distortion then is the amount of distortion as a function of the population size. This leads to an important question: at what stage do we say that enough distortion is present for the event to be caused by selection? If we make our detection criteria too lenient, then we increase the risk of type I errors (false positives), whilst stricter criteria increase the risk of type II errors (false negatives). What is the optimal place to draw this proverbial line in the sand when detecting segregation distortion?

The diversity of criteria used in the literature reflect the difficulty of answering this question. Some authors settle for a simple chi-square test with the minimum significance threshold of p < 0.05 [5,10,14–16], others report multiple significance thresholds [7,17–20], whilst others use corrections for multiple testing, including false discovery rate (FDR) [11,12,21] and the even stricter Bonferroni correction [4,22]. This inconsistency has the further implication that many of the studies on segregation distortion are not comparable, which is problematic for the general advancement of our knowledge of segregation distortion. It interferes with our ability to assess hypotheses such as: (i) levels of segregation distortion differ between different species, (ii) segregation distortion increases with the genetic distance between parents.

To circumvent the conflation between selection and chance, it would be useful if we could observe the processes that lead to the final genotypic composition of a RIL population. Whilst this is infeasible to achieve with real organisms, it is possible in an *in-silico* simulation of a RIL population. Here we utilize PedigreeSim [23], which computationally models single-nucleotide polymorphism (SNP) genotype data from a RIL population, starting with recombination between homologues during meiosis, generation of gametes and fusion of gametes to form a zygote. This process can be repeated for the desired number of filial generations. The simulation allows us to control multiple parameters that could influence segregation distortion, such as the number of SNP markers used, the position of selection in the genome, the strength of selection in the genome, the distribution of SNP markers, and the size of the population. We can also examine the interaction between different parameters, such as population size and selection. With knowledge of the parameters that produced the final genotyping dataset, we can then attempt to identify the appropriate threshold to detect segregation distortion by examining the performance of various statistical tests. For example, when a selection pressure of strength X is applied at a locus, in what proportion of simulated populations is this locus identified as being significantly distorted for a given statistical test and population size?

In addition to the simulation experiments performed, we also wanted to investigate how much of the purported segregation distortion typically observed in real populations is the result of random chance rather than a consistent selection pressure. To examine this, we produced replicate populations of the same cross between varieties Avalon and Cadenza. These consisted of two F2 populations with Avalon as the female parent, and two F2 populations with Cadenza as the female parent, with each population containing around 96 lines. We were then able to compare replicate populations and test whether they showed any consistency in the regions exhibiting segregation distortion, which if they did would imply that the distortion was the result of a selection pressure rather than random chance.

There is a trend in the literature of removing markers exhibiting segregation distortion before the construction of a genetic map [10,24,25]. It has already been shown by a previous simulation that segregation distortion does not affect the order of a genetic map [26]. High levels of segregation distortion can however effect the estimation of recombination frequency between a pair of markers [27], which is used in the clustering stage of genetic map construction. Here we use our simulation to examine whether clustering of markers is significantly affected by segregation distortion in modern genetic mapping software such as MSTMap [28].

Finally, after identifying appropriate statistical tests for the detection of segregation distortion from these experiments, we perform a reanalysis of some existing genotyping datasets from populations of hexaploid and tetraploid wheat [10,15]. This allows us to highlight important regions of segregation distortion that could be the subject of further investigation, potentially leading to the identification of the genomic position and mechanism of a causative locus of segregation distortion in wheat.

## Materials and methods

For the replicate empirical mapping populations, we generated F2 plants using bread wheat (*Triticum aestivum* L.) varieties Avalon and Cadenza in reciprocal crosses. All plants were grown in uniform conditions at the same time using pots filled with peat-based soil and kept in a glasshouse at 15–25 °C with 16-h light, 8-h dark. Leaf-tissue was harvested from F2 plants two weeks after sowing. DNA was extracted following the protocol in [29] with minor modifications.

DNA concentration was assessed using a Qubit 2.0 Fluorometer and was the normalized to 23 ng / μl ready for analysis with the Axiom^®^ Wheat Breeder’s array. Sample preparation for array genotyping was performed with the Beckman Coulter Biomek FX. Samples were then genotyped using the Axiom^®^ 35K Wheat Breeders array in conjunction with the GeneTitan^®^ using standard Affymetrix protocols (Axiom^®^ 2.0 Assay for 384 samples P/N 703154 Rev. 2).

### Genetic map construction

Axiom Analysis Suite (version 3.1.51.0) was used to assign genotype calls using the Axiom Best Practices Genotyping Workflow. There were 3044 SNPs polymorphic between the parental varieties, Avalon and Cadenza, that were deemed suitable for genetic mapping. These were designated as PolyHighResolution, which is the category assigned to markers that are clearly codominant, by Axiom Analysis Suite and had a minor allele frequency > 0.1. The minor allele frequency criterion served as a simple metric to remove markers with highly erroneous cluster plots from the analysis. Cluster plots of the probes that did not meet the minor allele frequency criterion were inspected by eye to ensure that no genuine cases of segregation distortion were omitted.

To create the genetic map, the genotyping data from the Cadenza X Avalon population was used. The ASMap package in R, an implementation of the MSTmap algorithm, was used for clustering, ordering and calculation of genetic distance between markers. Various values for the clustering parameter were tested during the creation of the genetic map. The final value used was 10^−25^, which returned several linkage groups that contained around 200 markers, which is in accordance with other genetic maps of wheat produced with the 35k Wheat Breeder’s array [10]. Chromosome assignment to linkage groups was based on information from nullisomic lines from CerealsDB [30] as well as a BLAST search of probe sequences against the IWGSC RefSeq v1.0 sequence [31] (hereafter referred to as the IWGSC assembly). Markers were assigned physical locations based on a BLAST search of probe sequences against the IWGSC assembly. Any linkage groups that spanned less than 80% of the physical distance of the chromosome were removed from the analysis, as we were interested in observing patterns of segregation along the entire length of the chromosome. Linkage groups representing the following chromosomes were retained: 1A, 1B, 1D, 2A, 2B, 2D, 3A, 3B, 4A, 4B, 5A, 5B, 6A, 6B, 7A.

### Simulation

Genotyping data from a single seed descent population were simulated using PedigreeSim [23] in conjunction with a custom wrapper script written in R [32]. The R script provides the capability to apply a selection pressure of a specified strength on gametes of a parental genotype at a locus. For example, we could apply a negative selection pressure of strength 1/20 at marker 200 against gametes with a “B” genotype, meaning that these gametes would be 5% less viable than gametes with an “A” genotype at this locus. We would therefore expect this selection pressure to produce a 100:95 ratio of A:B gametes. PedigreeSim allows the input of markers at specified centimorgan positions, meaning that we were able produce simulations that had the marker distribution of wheat chromosomes. For many of the simulations, we used the existing genetic map from the Cadenza X Avalon population to provide these marker positions so that the segregation distortion data were comparable to empirical populations of wheat. When performing simulations, we ran 1000 simulations for each unique set of parameter values unless otherwise stated.

To examine the effect of segregation distortion on genetic map construction, we simulated two chromosomes using the centimorgan positions from chromosomes 1A and 6B of the Cadenza X Avalon genetic map, which were chosen based on marker density. Before genetic map construction, the order of markers in the genotyping data was scrambled to ensure that this information was not being used by the mapping software. Firstly, we tested clustering when one selection pressure resulting in the highest level of distortion (0:0:1 ratio of AA:AB:BB genotypes) was applied to chromosome 1A at marker 200. We then tested clustering when each chromosome had a selection pressure applied at marker 30 and 200 of chromosomes 6B and 1A respectively in favour of the same parental allele. We also tested the effect of segregation distortion on map length using selection pressures of varying strengths at the positions previously mentioned.

To measure segregation distortion, we used a variety of methods. These include the magnitude of distortion, referred to here as *M*, which is defined as $\frac{a}{a+b}$ where a and b represent the number of plants with homozygous A and B genotypes respectively at an arbitrary locus. *M* ranges from 0 to 1, with 0 meaning no A genotypes are present and 1 meaning no B genotypes are present. For F2 populations, we use a chi-square goodness-of-fit test to measure deviation from a 1:2:1 ratio of AA:AB:BB genotypes, whilst for F6 populations, we measured deviation from a 1:1 ratio of AA:BB genotypes. Adjusted p-values were produced using the p.adjust function in R with either the Benjamini-Hochberg procedure for false discovery rate (FDR) correction or the Bonferroni correction.

## Results

### Validation of simulation

Simulated data closely resembled empirical data from the Cadenza X Avalon mapping population. The mean (± s.d.) number of crossover events per plant for chromosome 1A was 2.72 ± 3.31, and 2.59 ± 1.31 in empirical and simulated populations respectively, each population containing 96 individuals. There was no significant difference between the number of crossover events in individuals between empirical and simulated data as determined by a Mann-Whitney U test (p = 0.07). The mean (± s.d.) length of simulated genetic maps over 1000 simulations, using 96 individuals and the marker distribution from chromosome 1A of the Cadenza X Avalon genetic map, was 130.9 ± (7.3) centimorgans (cM), whilst the length of the empirical map was 130.48 cM. Simulated data closely conformed to the expected levels of heterozygosity for each filial generation (which should reduce by half for each generation in selfing organisms), with mean (± s.d.) values over 1000 simulations of 49.96 (± 1.73)%, 25 (± 1.42)%, 12.47 (± 0.97)%, 6.24 (± 0.7)%, 3.13 (± 0.48)% for F2, F3, F4, F5 and F6 generations respectively.

**Fig 1:**
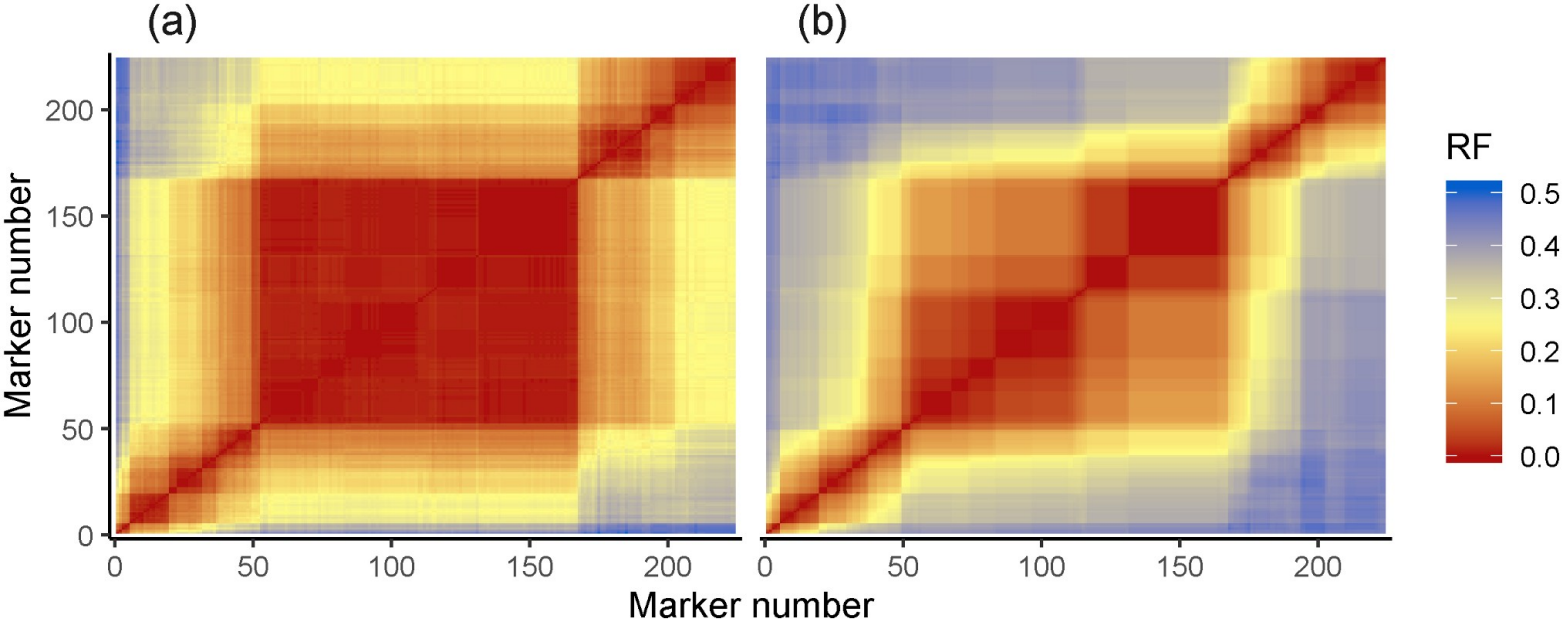
Comparison of recombination fraction heatmaps for both empirical (a) (Avalon X Cadenza 1A) and simulated data (b). The large central red block most likely represents the centromeric region of the chromosome, as wheat is known to have a lack of recombination in this area. The pattern of recombination cold spots (represented by red squares) is largely conserved between empirical and simulated data. The empirical data has low to medium levels of recombination between distant markers (represented by yellow regions), whilst the simulated data shows high levels of recombination (represented by blue regions).

**Fig 2:**
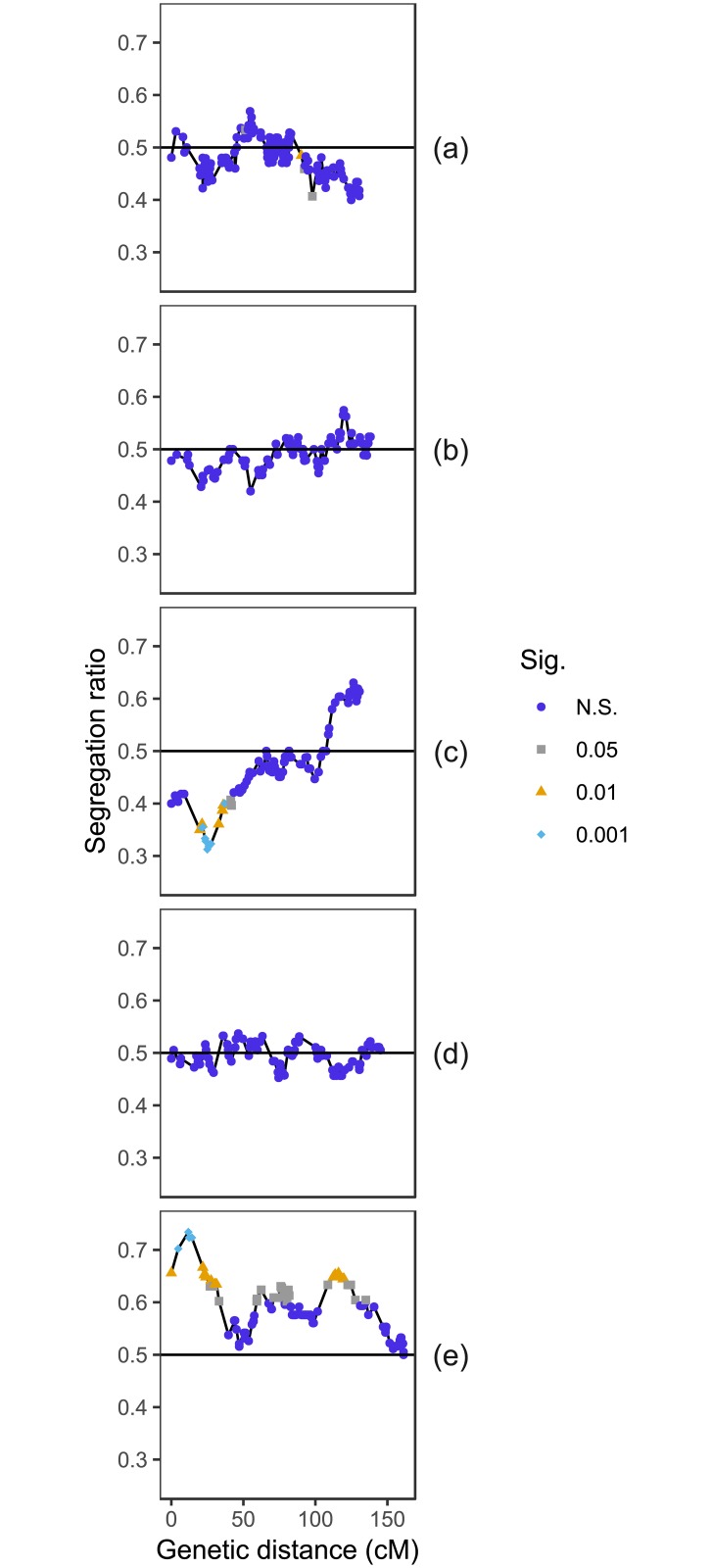
Comparison of empirical data (a) from chromosome 1A of a Cadenza X Avalon F2 mapping population with 96 individuals to simulated data (b–d). The simulations each have 96 individuals and were produced using the marker distribution from the empirical data. The y-axis shows the segregation ratio of homozygous genotypes, shown here as a proportion of the total number of homozygous genotypes $\left(\frac{a}{a+b}\right)$. The black horizontal line indicates an even 1:1 ratio of homozygous genotypes. Included are simulations of both F2 **(b, c)** and F6 **(d, e)** single seed descent populations for comparison, as well as simulations exhibiting the least **(b, d)** and the most **(c, e)** amount of segregation distortion out of 1000 simulations. None of the simulations have any selection pressure applied, so these plots indicate the effect of sampling error on segregation. Sig. = significance threshold (chi-square test).

In the recombination frequency heatmaps (Fig 1) of empirical and simulated data, the regions of low recombination between closely linked markers along the diagonal are largely preserved, whilst in the simulation, recombination frequency rises faster than in the empirical data with increasing distance between markers. This is to be expected, as recombination frequency (or a proxy measure, in this case the hamming distance for MSTmap) is used in the clustering stage of genetic map construction, meaning we do not expect to see pairs of markers above a particular recombination frequency threshold together in a single linkage group of the empirical data. Segregation of genotypes across markers in the simulated data are more autocorrelated than in empirical data, with values of 0.95 ± 0.03 (averaged over 1000 simulations with populations of 96 individuals and no selection) and 0.875 respectively (Fig 2). This is expected as the empirical data contains both genotyping errors and missing data whilst the simulated data does not.

### Simulation experiments

**Fig 3:**
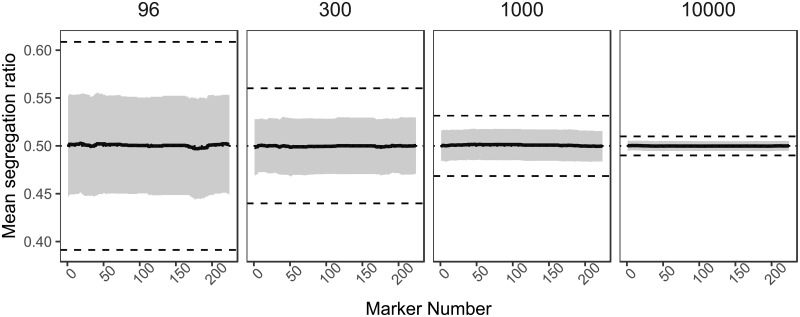
Effect of population size on the magnitude of distortion. Indicated in the header of each panel is the population size. Segregation ratio is calculated as $\frac{a}{a+b}$, and each data point is the mean value over 1000 simulations. The simulations have no selection and use the marker distribution from chromosome 1A of the Cadenza x Avalon cross. The shaded area represents the mean segregation ratio value ± the standard deviation over 1000 simulations. The dashed lines mark the 5% significance threshold for a chi-square test, whilst the dotted line marks a 1:1 segregation ratio. The effect of sampling error on segregation ratio decreases as population size increases.

Initially, we ran simulations using the marker distribution for chromosome 1A of the Cadenza X Avalon cross for population sizes of 96, 300, 1000 and 10000, all with 224 markers and no selection applied. *M* decreased with increasing population size, whilst the proportion of simulations that contained markers exhibiting significant segregation distortion stayed relatively constant, as shown in Table 1. To test the effect of marker binning on the detection of segregation distortion, simulated genotyping datasets with a reduced marker set (93 markers) containing only skeleton markers were produced. Only the FDR and Bonferroni corrections showed any differences between marker sets (Table 1). Increasing population sizes decrease the variance in segregation between simulations, but also make chi-square significance criteria more sensitive (Fig 3). Filial generation did not influence the number of simulations that exhibited significant segregation distortion (Table 1) according to a chi-square test, (comparison of F2 and F6 with population size 300, χ^2^ = 0.004, df = 1, p = 0.95).

**Table 1:** Measures of segregation distortion for simulations with 224 markers and marker distribution taken from chromosome 1A of a Cadenza X Avalon F2 cross. The last column indicates the mean value across all simulations of the magnitude of distortion at its highest value. Shown in the p-value columns are the number of simulations (out of 1000 performed) that contain significantly distorted markers. Marker set A refers to the full marker set of 224 markers, whilst marker set B refers to the skeleton marker set of 93 markers.

Population Size	Marker set	Filial Generation	P < 0.05	P < 0.01	P < 0.001	P < 0.05 (FDR Correction)	P < 0.05 (Bonferroni Correction)	Mean magnitude of peak distortion
96	A	2	561	162	16	18	4	0.141451
300	A	2	561	163	28	26	4	0.08122
1000	A	2	557	177	24	27	7	0.043656
10000	A	2	557	183	28	28	6	0.013773
96	A	6	602	179	27	30	7	0.109901
300	A	6	563	167	19	33	1	0.061526
1000	A	6	570	215	35	38	5	0.034302
10000	A	6	583	208	26	28	3	0.010845
96	B	2	561	162	16	22	8	0.141451
300	B	2	561	163	28	25	13	0.08122
1000	B	2	557	177	24	26	15	0.043656
10000	B	2	557	183	28	29	17	0.013773

In simulations containing a single marker for a population size of 1000, 5.2% exhibited significant segregation distortion, which is concordant with a chi-square alpha threshold of 0.05. Where two markers were placed near each other at centimorgan positions of 59 and 60, 5.4% of simulations contained markers with significant segregation distortion. Increasing the distance between these two markers by placing them at 20 and 60 centimorgans resulted in 9.2% of simulations containing markers with significant segregation distortion. The difference in number of simulations containing segregation distortion between these proximal and distal marker distributions was significant (χ^2^ = 9.89, df = 1, p = 0.002).

**Fig 4:**
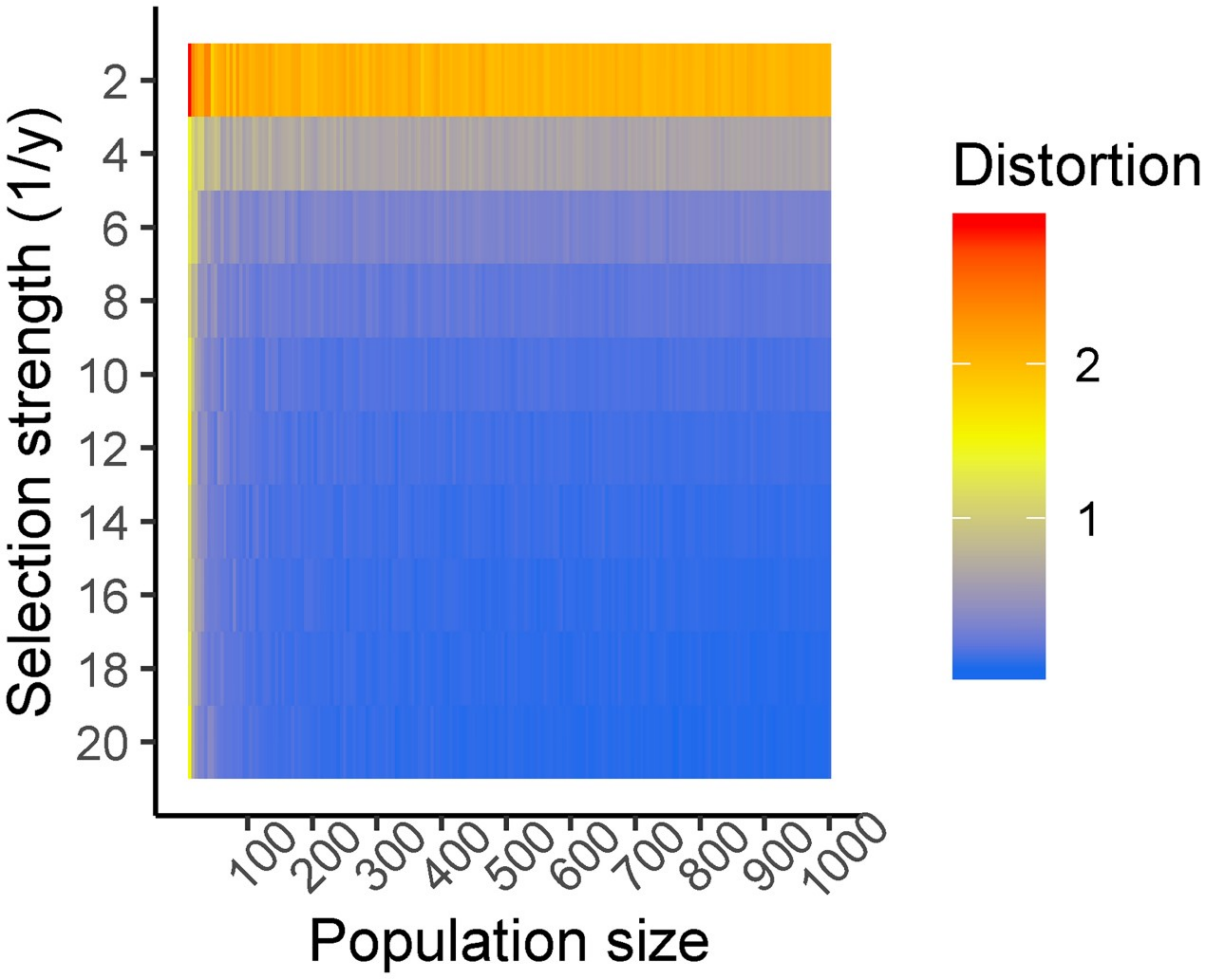
Heatmap of deviation from 1:1 segregation of homozygotes at various population sizes and selection strengths. Lower selection strengths are highly dependent on population size. As population size decreases, the influence of sampling error on segregation ratios increases, leading to high segregation distortion even in the case of weak selection. Each tile is an average value over 20 simulations with 20 markers at evenly spaced intervals, totalling 100 centimorgan. Deviation is calculated as ∑(*y* − 0.5)^2^ where y is the ratio of homozygous genotypes $\frac{a}{a+b}$ at an arbitrary locus; a is the number of homozygous genotypes from parent 1, b is the number of homozygous genotypes from parent 2 at an arbitrary locus.

To assess the effects of population size and selection strength on deviation from a 1:1 ratio of homozygous genotypes, and therefore segregation distortion, we ran a set of simulations in which both these parameters varied (Fig 4). Population size ranged from 10 to 2000, whilst selection strength ranged from 1/20 to **½**. As selection strength increases, the effect of population size on the deviation from 1:1 decrease. Simulations with population sizes less than 80 are very susceptible to distortion regardless of the selection strength.

**Fig 5:**
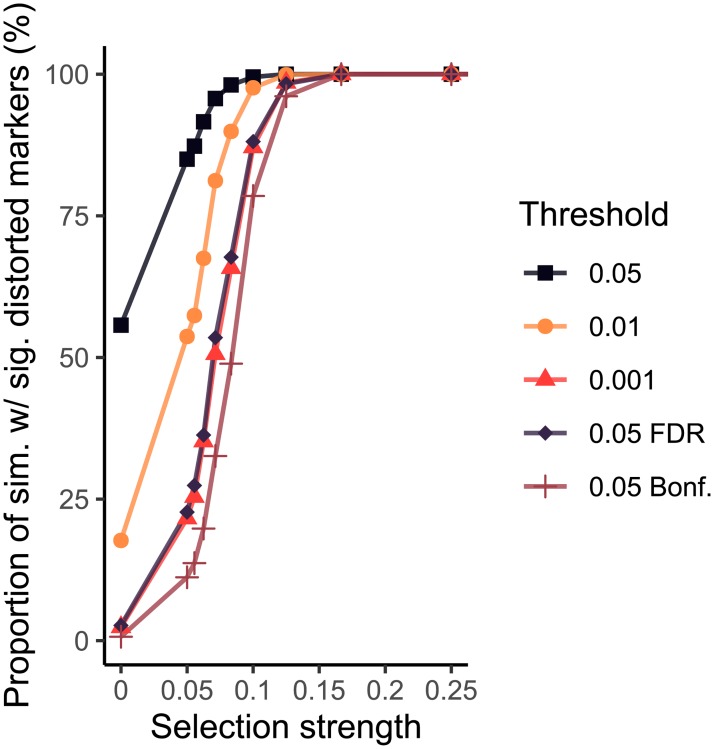
Proportion of 1000 simulations containing significantly distorted markers as a function of selection strength for various p-value threshold criteria. Simulations contain 1000 individuals and used the marker distribution of chromosome 1A from the Cadenza X Avalon F2 population. The position of selection was at locus 200 of 224 markers Sim = simulations, sig. = significant, pop. = population.

We examined the performance of various p-value thresholds and multiple testing procedures on the detection of segregation distortion at a range of selection strengths, with a population of 1000 individuals per simulation (Fig 5). 56.1% of simulations contained significantly distorted markers when a p-value threshold of 0.05 was used with no selection, compared to 3% at p-value thresholds of p < 0.001, p < 0.05 (FDR corrected) and p < 0.05 (Bonferroni corrected). As shown in Fig 5, p < 0.001 and p < 0.05 (FDR corrected) are almost equivalent for this distribution of markers. All the detection criteria reach saturation (100% of simulations having markers with significant segregation distortion) at a selection strength of 0.25. As expected, the Bonferroni test is strictest regardless of selection strength.

**Fig 6:**
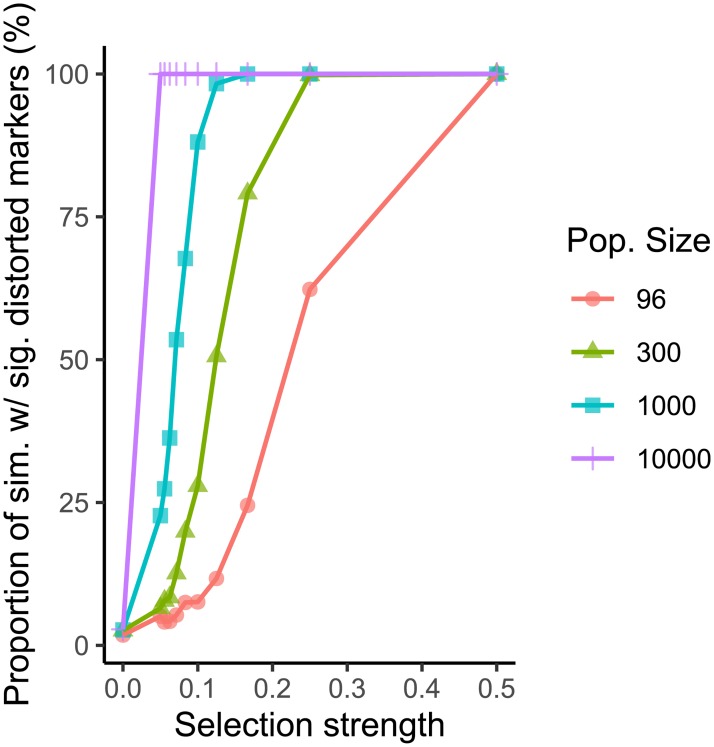
Effect of selection strength and population size on the number of simulations containing distorted markers (as determined by a chi-square test with significance threshold of 0.05 after correction for multiple testing with FDR). Sim = simulations, sig. = significant, pop. = population.

In addition to the type of statistical test used, population size also influences the number of simulations exhibiting significant segregation distortion. As population size increases, so does the ability to reliably detect smaller selection strengths (Fig 6). At a selection strength of 1/20, 60.3%, 66.2%, 85% and 100% of simulations contained markers exhibiting significant segregation distortion under a chi-square test with alpha threshold 0.05 for population sizes of 96, 300, 1000 and 10000 respectively (S1(a) Fig).

To test whether local recombination rate in the region of selection effected the detection of segregation distortion, we performed additional simulations with selection at marker 100 of chromosome 1A. This marker is located in a region of low recombination (S2 Fig), which contrasts with previous simulations where selection was at marker 200, located in a region of high recombination. The only statistical test that was affected by recombination rate in the region of selection was the FDR correction, which was consistently more powerful at all values of selection strength for population sizes 96, 300 and 1000 (S3 Fig).

These results contrast with the effect of local recombination rate on the detection of segregation distortion regions (SDRs), defined here as 2 or more consecutive markers exhibiting significant segregation distortion. The total number of SDRs generated among all 1000 simulations is generally higher when selection is positioned at marker 100 compared to marker 200 (S4(a) Fig). An exception to this occurs at very high selection strengths (0.5), as these cause the entire chromosome to become one SDR at position 100, resulting in fewer, albeit larger in number of markers, SDRs overall. Changing the measure of SDRs to the number of simulations containing at least one SDR (S4(b) Fig) causes both selection positions to perform almost analogously when using no multiple testing correction and a detection threshold of p < 0.001. In comparison, the FDR correction for multiple testing results in a greater difference between positions.

To examine the effect of selection strength on the position of maximum distortion, we performed simulations with a selection pressure at locus 200 of 224, using the marker distribution from Cadenza X Avalon 1A and a population size of 300 individuals. Selection strength ranged from 1/20 to ½. As expected, the number of markers exhibiting significant segregation distortion increased with the selection pressure, with mean values of 30.83 and 177.45 at selection pressures of $\frac{1}{20}$ and ½ respectively. The percentage of simulations at which the peak of segregation distortion occurred within 10 markers of the applied selection pressure was52% for a selection pressure of $\frac{1}{10}$, which gradually increased: 64.9%, 78.2%, 92.7% and 99.7% for pressures of $\frac{1}{8},\frac{1}{6},\frac{1}{4}$, and ½ respectively. This was also affected by population size, with higher population sizes having an increasing number of simulations exhibiting peak segregation distortion at the selection locus when selection strength was fixed to 1/20 (S1(b) Fig).

### Effect of segregation distortion on genetic mapping

**Fig 7:**
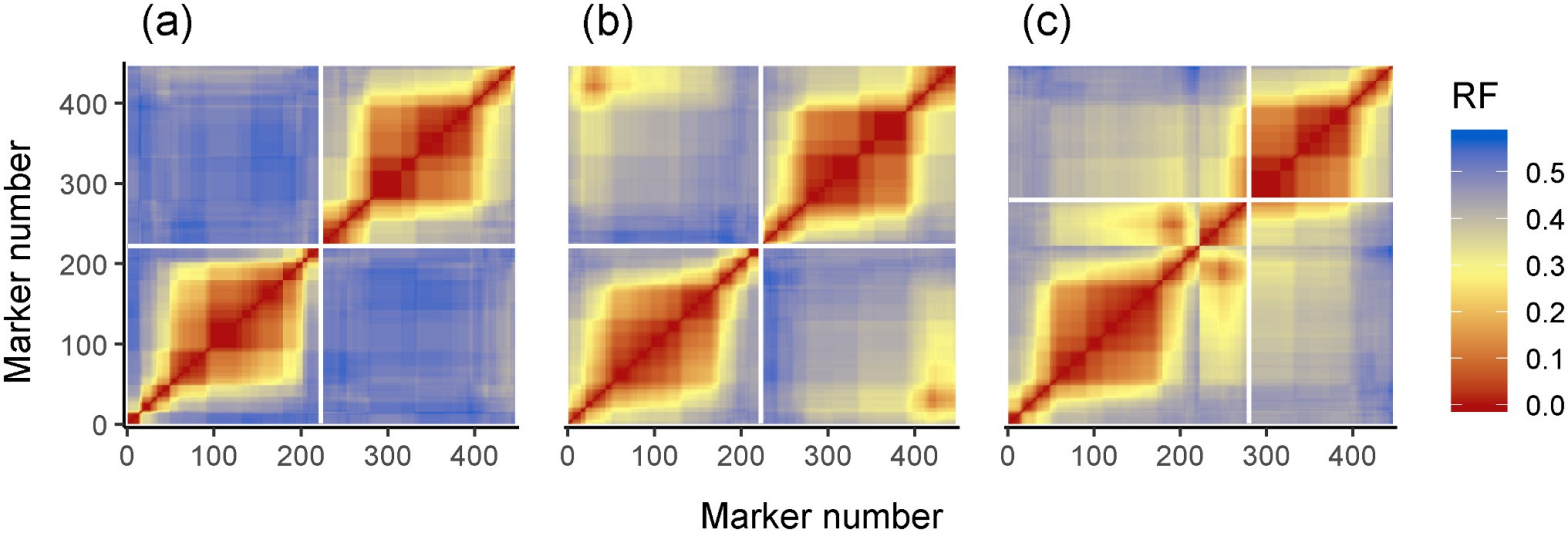
Effect of segregation distortion on genetic mapping. **(a)** When no selection is applied, two linkage groups representing simulated chromosome 1A and chromosome 6B are formed with perfect order of marker bins. **(b)** When a strong selection pressure of $\frac{1}{1.11}$ is applied at locus 30 and 200 of 6B and 1A respectively, the recombination fractions between markers at these loci and surrounding markers are lowered, but not enough to disrupt clustering or ordering of the markers. **(c)** When a strong enough selection pressure of $\frac{1}{1.105}$ is applied such that one of the loci has zero A genotypes, the recombination frequencies of markers under selection are low enough such that chromosomal fragments experiencing segregation distortion are concatenated into the same linkage group. White lines delineate linkage groups.

In the simulations with a single selection pressure of strength 1 at locus 30 of chromosome 6B and no selection pressure on 1A, yielding a genotype ratio of 0:0:300 at the locus under selection, MSTMap was able to construct the genetic map with perfect clustering and ordering of marker bins using a clustering parameter of 10^−43^. The second simulation contained two selection pressures, one positioned at marker 30 of chromosome 6B and the other positioned at marker 200 of chromosome 1A, both favouring the same parental genotype. MSTMap was able to construct genetic map with perfect clustering and ordering of marker bins (using clustering parameter 10^−45^) up to a selection pressure of $\frac{1}{1.11}$, which yielded genotype ratios of 1:31:268 (test of deviation from 1:2:1 ratio: χ^2^ = 664.07, df = 2, p < 10^−15^) and 2:32:266 (test of deviation from 1:2:1 ratio: χ^2^ = 650.29, df = 2, p < 10^−15^) for the markers under selection respectively. When the selection strength for this configuration was increased to $\frac{1}{1.105}$, yielding genotypes ratios of 0:27:273 and 1:28:271 respectively, MSTMap was unable to cluster markers correctly for any of the clustering parameters tested, which ranged from 10^−40^ to 10^−50^. For example, using a clustering parameter of 10^−45^ yielded two linkage groups, the first consisting of markers 1 to 167 of chromosome 1A, the second consisting of a concatenation of 1A markers 168 to 223 and all the markers on 6B (Fig 7).

Moving the position of one of the two selection pressures from a region of high recombination (marker 200 on chromosome 1A, S2 Fig) to a region of low recombination (marker 100 on chromosome 1A, S2 Fig), had little effect on genetic map construction, with MSTMap producing a map with perfect clustering and ordering of marker bins at a selection strength of 1/1.2, yielding genotype ratios of 2:50:248 and 2:55:243 at marker 30 of chromosome 6B and marker 100 of chromosome 1A respectively. MSTMap was unable to cluster markers correctly when selection strength was increased to 1/1.1, yielding genotype ratios of 0:29:271 and 0:31:269 for the respective markers under selection. When the position of selection on chromosome 6B was also moved to a region of low recombination (from marker 30 to marker 110, S5 Fig), MSTMap produced a map with perfect clustering and ordering of marker bins at a selection strength of 1/1.2, and failed to cluster markers correctly when the strength was increased to 1/1.1.

Similarly to the results from the F2 population, in a simulation of an F8 SSD population with the same selection positions and a selection strength of $\frac{1}{1.3}$, which yielded AA:BB genotype ratios of 28:272 (chi-square test of deviation from 1:1 ratio, χ^2^ = 198.45, df = 1, p < 10^−15^) and 31:269 (chi-square test of deviation from 1:1 ratio, χ^2^ = 188.81, df = 1, p < 10^−15^) for the respective markers under selection, MSTmap was able to produce perfect clustering and ordering of marker bins with a clustering parameter of 10^−42^.

Extreme segregation distortion caused a significant shortening of map length for simulated chromosome 6B (t-test, t = -27.57, df = 176.43, p < 10^−15^) by around 20 cM, with a selection pressure of 1 producing a map length of 140.14 ± 3.92 compared to 159.07 ± 5.64 with no selection applied. Less extreme selection pressures of 1/3, 1/5, 1/7 and 1/9 produced mean map lengths over 100 simulations of 153.04 ± 4.64, 155.08 ± 4.4, 155.39 ± 4.68 and 156.77 ± 3.95 respectively. Likewise, for simulated chromosome 1A, extreme distortion shortened the map length significantly (t-test, t = -5.17, df = 197.72, p < 10^−6^), but with a smaller effect size than for 6B, with selection pressure of 1 producing a mean map length of 127.39 ± 4.81 compared to 130.84 ± 4.64 cM with no selection.

### Reanalysis of existing data

We reanalysed data from [10,15], both of which used the minimum chi-square threshold of p < 0.05 to detect regions of segregation distortion (Table 2). As expected, in both cases we observe a large reduction in the number of markers exhibiting significant segregation distortion when corrections for multiple testing are applied.

**Table 2:** Reanalysis of genotyping data from existing studies with corrections for multiple testing. Indicated in columns 3–5 are number of markers exhibiting significant segregation distortion with no correction for multiple testing, the FDR correction and the Bonferroni correction respectively.

Author	Mapping Population	P < 0.05	P < 0.05 (FDR)	P < 0.05 (Bonferroni)
Allen et al., 2016	Avalon X Cadenza	487	5	5
‘’	Savannah X Rialto	230	0	0
‘’	Opata X Synthetic	346	0	0
‘’	Apogee X Paragon	320	35	21
‘’	Chinese Spring X Paragon	774	0	0
Avni et al., 2014	Svevo X Zavitan	3789	1771	150

Markers that were still classified as significantly distorted in the Avalon X Cadenza population under Bonferroni correction were located on chromosomes 2A and 2D, whilst in the Apogee X Paragon population these were found on chromosomes 2D, 3B, 6A and 6B. Likewise, for the Svevo X Zavitan population, markers still significantly distorted under Bonferroni correction were found on chromosomes 2B and 3B.

### Cadenza X Avalon replicates

In the Cadenza X Avalon F2 replicates, there are 453 (14.88%) markers that exhibit significant segregation distortion (p < 0.05) in at least one of the replicates. Only 14 markers showed significant segregation distortion in both replicates. When both datasets were combined, 253 markers showed significant distortion. In the combined dataset, 173 of the 253 distorted markers were also distorted in one of the two original replicate datasets, whilst 80 were not. In the first and second replicates, 187 and 280 markers exhibited significant distortion respectively.

In the first replicate, there were 22 SDRs, comprised of 161 markers in total. The mean ± sd length of the SDRs was 7.32 ± 5.57 markers. In the second replicate, there were 31 SDRs comprised of 238 markers in total. The mean ± s.d. length of the SDRs was 7.68 ± 10.46. Three of the SDRs on chromosomes 1D, 5B and 1A respectively overlapped between replicates; the lengths of the overlaps were 4, 2 and 2 markers respectively.

### Avalon X Cadenza replicates

In the Avalon X Cadenza F2 replicates, there are 510 (16.75%) markers that exhibit significant segregation distortion (p < 0.05) in at least one of the replicates. Only 38 markers showed significant segregation distortion in both replicates. When both datasets were combined, 173 markers showed significant distortion. In the combined dataset, 120 of the 173 distorted markers were also distorted in one of the two original replicate datasets, whilst 53 were not. In the first and second replicates, 193 and 355 markers exhibited significant distortion respectively.

**Fig 8:**
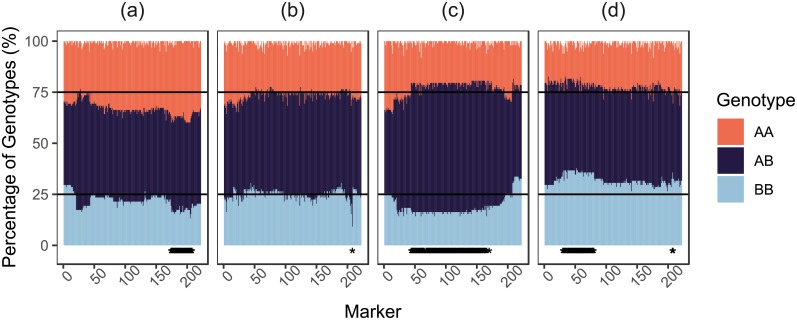
Comparison of segregation ratios on chromosome 6B for Cadenza X Avalon (a, b) and Avalon X Cadenza (c, d) replicates. Markers exhibiting significant segregation distortion as determined by a chi-square goodness of fit test for deviation from a 1:2:1 ratio of AA:AB:BB genotypes are highlighted by asterisks at the base of each plot. Black horizontal lines mark the expected transition from one genotype to the next under a 1:2:1 ratio. Markers are ordered on the x-axis as in the genetic map produced from the first replicate Cadenza X Avalon population.

In the first replicate, there were 15 SDRs comprised of 155 markers total. The mean ± s.d. length of the segregation distortion regions was 10.33 ± 8.81. In the second replicate, there were 20 SDRs comprised of 328 markers total. The mean ± s.d. length of the segregation distortion regions was 16.4 ± 32.49. Six of the SDRs overlapped between replicates, these were all located on chromosome 6B and had widths of 8, 4, 8, 2, 4 and 4 markers respectively, (Fig 8). The overlapping region did not have a skew towards the same parental genotype in each replicate.

## Discussion

Comparisons between the simulated data and the empirical data from the Cadenza X Avalon F2 mapping population show that the simulator is accurate in terms of map length produced, number of recombination events per individual, degree of segregation distortion and recombination frequency heatmap. We can therefore be confident that the subsequent simulation experiments are an adequate representation of reality.

It is clear *a priori* that when we test for segregation distortion, the probability of a false-positive result increases with the number of markers, as multiple inferences are being made simultaneously. What complicates the calculation of how much more likely a false-positive result is with increasing number of markers, and therefore how strict our correction for multiple testing should be, is the marker distribution along the chromosome. All markers are ultimately linked together to varying degrees by the process of recombination, so not all the statistical tests performed are completely independent. Markers adjacent to each other at short genetic intervals along the chromosome segregate in a highly linked manner. This is confirmed by our simulation experiments with different distributions of two markers, one in which the markers are close together and one in which they are further apart, with the latter yielding almost double the amount of simulations containing markers that were significantly distorted. Interestingly, the chi-square test performs as expected when only one marker is used on the chromosome with no selection, with around 5% of simulations showing a false-positive result, which corresponds to the traditional alpha threshold of 0.05.

The FDR correction procedure reassuringly produced an alpha threshold that is only slightly stricter than the traditional 5% in the simulated chromosome 1A described earlier, as shown in Table 1. The Bonferroni correction is only appropriate when statistical tests are completely independent from one another, which is not the case for highly linked markers. The Bonferroni test would therefore be appropriate if markers were on different chromosomes, or if they were located at large distances from each other on the same chromosomes. For high-density SNP data obtained from microarrays this is often not the case, and therefore the Bonferroni correction is often too strict, as shown by the results in Table 1, where in the F2 population of 300 individuals only 4 simulations show significant segregation distortion, where we would expect around 50 if the test corresponded to the usual 0.05 alpha threshold. The fact that 56% of simulations without any selection pressure show significant segregation distortion according to the chi-square test at the minimum p-value threshold (p < 0.05) should definitively rule out the use of chi-square without correction for multiple testing, or inclusion of lower thresholds, in future studies that utilize high-density genotyping data. The perfect multiple testing procedure for segregation distortion would be one that considers the distribution of markers on the chromosome such that the alpha threshold is adjusted depending on the degree of linkage between each marker. However, seeing as the FDR correction for multiple testing is only marginally more conservative than the traditional alpha threshold of 0.05, and taking into account the fact that the traditional alpha threshold was chosen arbitrarily [33], the use of FDR as a new standard for the detection of segregation distortion seems appropriate.

One focus in the literature is the identification of segregation distortion loci. Our simulation experiments with a range of selection strengths show that resolution of selection events is increased with population size. This is because the effects of sampling error are neutralized as population size increases. Sampling error could lead to the erroneous conclusion that a segregation distortion locus is present by shifting the segregation of a marker away from expected mendelian ratios. Conversely, it can make markers under true selection pressures appear as normally segregating. It can also skew the peak of segregation distortion away from a true selection locus at smaller selection strengths. To correctly identify the causative locus in this case then would require a wider search than is initially implied by the segregation data. These results emphasize the significance of sampling error in segregation distortion studies. In addition, our results show that local recombination rates in the region of selection have little influence on the detection of segregation distortion.

It has long been known that segregation distortion effects the estimation of recombination fraction between markers [27]. However, there does not seem to be a practical guide in the literature that can assist researchers in knowing what degree of distortion will affect the mapping process. Our simulation experiments on the effect of segregation distortion on genetic mapping show that only very extreme distortion effects the formation of linkage groups during the clustering of markers, as well as map length, meaning that markers experiencing moderate distortion can be retained in genetic maps. This conclusion persists regardless of selection position, whether in a region of high or low recombination. This result will be useful to future studies, as markers that would have previously been discarded will give us more information on potentially useful genomic regions of many crop species.

In the re-analysis of data from Allen et al [10] and Avni et al [15], it is interesting to note that the latter had many more markers exhibiting segregation distortion both before and after corrections for multiple testing. The former study used varieties of bread wheat (*Triticum aestivum* L.) whilst the latter involved a cross between durum wheat (*Triticum turgidum* L. *subsp*. *durum*) and a wild relative of durum wheat, wild emmer (*Triticum turgidum* L. *subsp*. *dicoccoides*). It has been noted elsewhere in the literature that the degree of segregation distortion often increases with genetic distance of the parents [34]. One hypothesis is that with increasing genetic distances, the fitness benefits conferred to the progeny of biparental crosses become increasingly different between parental alleles. If this is indeed the case, the description of a true segregation distortion locus in closely related crop varieties, including its mechanism of action, is a much more difficult task than in more distantly related crosses. Indeed, our best descriptions of segregation distortion loci are from crosses between rice (*Oryza sativa* L.) subspecies *indica* and *japonica* [35], as well as *Drosophila pseudoobscura* subspecies *pseudoobscura* and *bogotana* [2]. To detect a true segregation distortion locus in closely related wheat varieties then would require population sizes large enough to detect much smaller selection strengths, as indicated in Fig 6, as well as replicate populations to confirm the effect on segregation is due to selection. An exception to this statement may be in the production of doubled haploid mapping populations, where differences in amenability to doubled haploidy between closely related varieties has the potential to produce segregation distortion that is stronger than in an SSD population structure [36].

When identifying segregation distortion in empirical populations, it is often convenient to assess segregation in terms of SDRs, as multiple consecutive markers exhibiting significant segregation distortion provide us with more confidence that the distortion observed is not due to erroneous genotype assignment. The fact that we only observed a few SDR overlaps that were distorted towards the same parent between replicates in our empirical populations shows that legitimate segregation distortion between varieties of wheat is rare. Our simulation experiments also confirm the intuitive deduction that the number of SDRs should increase when selection occurs within regions of low recombination.

In conclusion, the results presented here emphasize the importance of using appropriate statistical methods when detecting segregation distortion. We must be sure that the observed distortion is due to a genuine selection pressure before we can commence further research into identifying the loci that are driving the distortion. We recommend that studies utilizing high-density genotyping data use an FDR correction for multiple testing when checking for segregation distortion, and that population size should be as high as possible to increase the chances of discovering genuine segregation distortion loci. Fig 6 serves as a guide for the appropriate population size to detect various selection strengths. For example, to reliably detect a selection strength of $\frac{1}{10}$ at the 0.05 p-value threshold, a population size slightly bigger than 1000 individuals is required. As a result of our reanalysis of existing data based on these principles, we have discovered a candidate segregation distortion region on chromosome 3B of a cross between wheat varieties Apogee and Paragon that is likely to be caused by a genuine selection event. We hope that future studies of segregation distortion will also consider the findings presented here.

## Supporting information

S1 Fig Simulation of an F5 RIL population with a selection pressure of strength 1/20 at locus 200. Indicated in the header of each panel is the population size. As the population size increases, the influence of sampling error on segregation of marker decreases, providing increased resolution of genuine selection events. (a) shows the mean magnitude of distortion ((a)/(a + b)) over 1000 simulations. The shaded area represents ± the standard deviation of the magnitude of distortion over 1000 simulations. The dashed lines mark the 5% significance threshold for a chi-square test, whilst the dotted line marks a 1:1 segregation ratio. (b) shows the number of simulations in which the peak of distortion occurs at the specified marker. As population size increases, so do the number of simulations in which the genuine selection event emerges as the peak of distortion. Num. = Number, sim. = simulations, dist. = distortion.
(PDF)

S2 Fig Recombination for chromosome 1A of the Avalon X Cadenza cross. The amount of recombination is represented by the slope of the line.
(PDF)

S3 Fig Examining the effect of selection position on the number of simulations containing significantly distorted markers. Position 100 is in a region of low recombination, whereas position 200 is in a region of high recombination. Columns are separated by the type of statistical test performed, whereas rows are separated by population size. The FDR correction has consistently more power when selection is in position 100. sig. = significant, dist. = distorted, S. = selection, Pos. = position.
(PDF)

S4 Fig Comparison of the effect of selection position on formation of segregation distortion regions (SDRs). Selection position 100 is in a region of low recombination, whereas position 200 is a region of high recombination. (a) shows the total number of SDR among 1000 simulations as a function of selection strength, whereas (b) shows the number of simulations with at least 1 SDR. Shown in the panel titles are the thresholds / type of statistical tests used to detect segregation distortion. num. = number, sim. = simulations.
(PDF)

S5 Fig Recombination for chromosome 6B of an Avalon X Cadenza cross. The amount of recombination is indicated by the slope of the line.
(PDF)

S1 Table Genotyping data and genetic map for the Avalon X Cadenza reciprocal crosses. (CSV)

S1 File R scripts implementing selection for PedigreeSim. (ZIP)
