# Supplementary material for: Segregation distortion: Utilizing simulated genotyping data to evaluate statistical methods
Source: PLoS One. 2020 Feb 19;15(2):e0228951. doi: 10.1371/journal.pone.0228951 (PMC7029859; doi:10.1371/journal.pone.0228951)

S1 Fig. Simulation of an F5 RIL population with a selection pressure of strength 1/20 at locus 200. Indicated in the header of each panel is the population size. As the population size increases, the influence of sampling error on segregation of marker decreases, providing increased resolution of genuine selection events. **(a)** shows the mean magnitude of distortion ((a)/(a + b)) over 1000 simulations. The shaded area represents ± the standard deviation of the magnitude of distortion over 1000 simulations. The dashed lines mark the 5% significance threshold for a chi-square test, whilst the dotted line marks a 1:1 segregation ratio. **(b)** shows the number of simulations in which the peak of distortion occurs at the specified marker. As population size increases, so do the number of simulations in which the genuine selection event emerges as the peak of distortion. Num. = Number, sim. = simulations, dist. = distortion

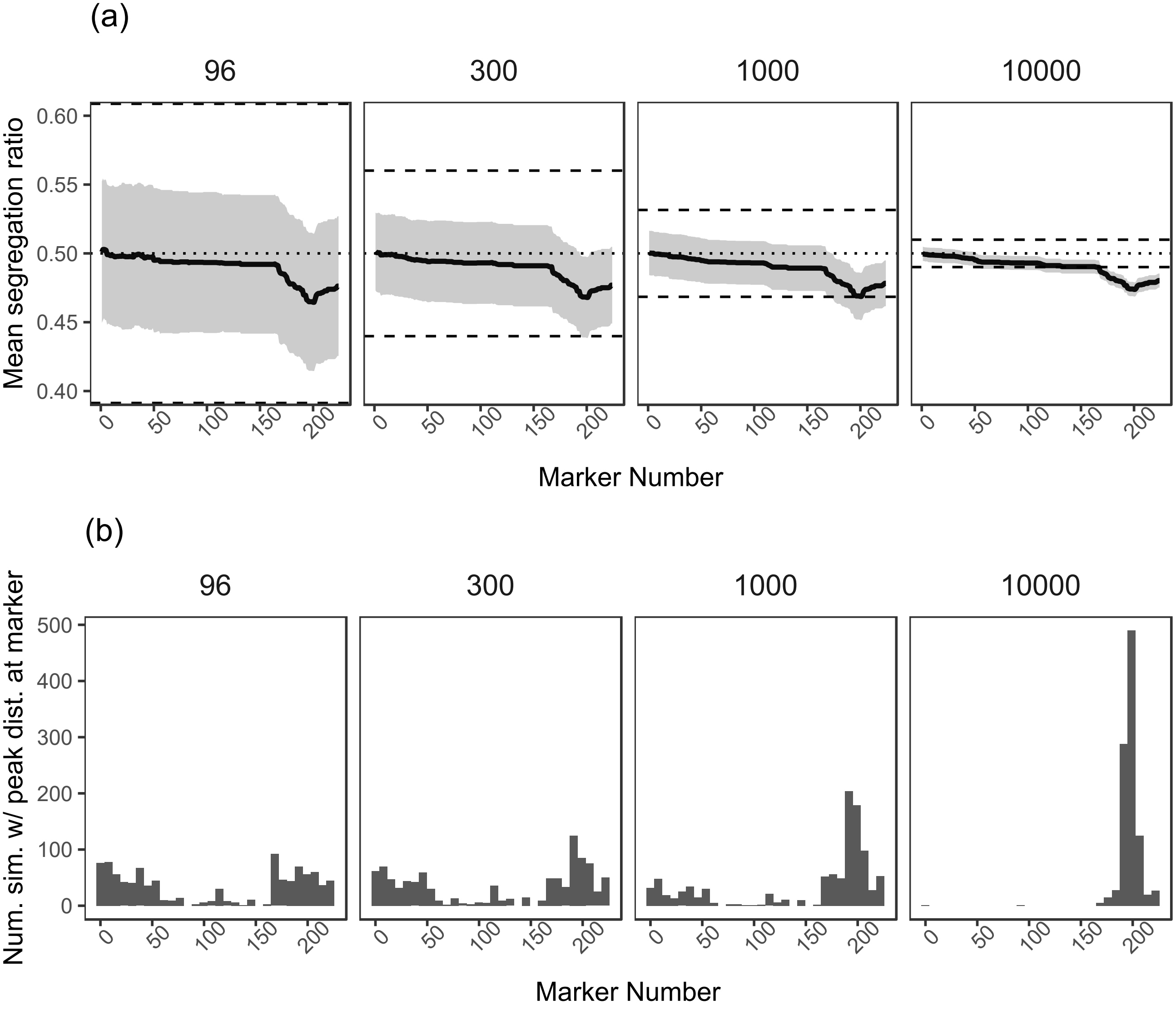

Supplement: S1 Fig — Indicated in the header of each panel is the population size. As the population size increases, the influence of sampling error on segregation of marker decreases, providing increased resolution of genuine selection events. (a) shows the mean magnitude of distortion ((a)/(a + b)) over 1000 simulations. The shaded area represents ± the standard deviation of the magnitude of distortion over 1000 simulations. The dashed lines mark the 5% significance threshold for a chi-square test, whilst the dotted line marks a 1:1 segregation ratio. (b) shows the number of simulations in which the peak of distortion occurs at the specified marker. As population size increases, so do the number of simulations in which the genuine selection event emerges as the peak of distortion. Num. = Number, sim. = simulations, dist. = distortion. (PDF) [file pone.0228951.s001.pdf]
