# Supplementary material for: Segregation distortion: Utilizing simulated genotyping data to evaluate statistical methods
Source: PLoS One. 2020 Feb 19;15(2):e0228951. doi: 10.1371/journal.pone.0228951 (PMC7029859; doi:10.1371/journal.pone.0228951)

S2 Fig. Recombination for chromosome 1A of the Avalon X Cadenza cross. The amount of recombination is represented by the slope of the line.

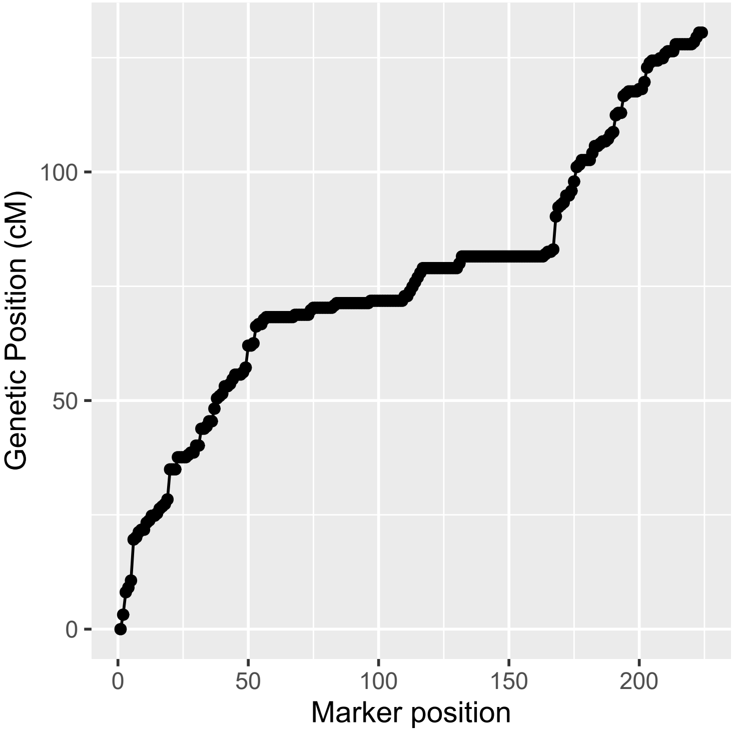

Supplement: S2 Fig — The amount of recombination is represented by the slope of the line. (PDF) [file pone.0228951.s002.pdf]
