# Supplementary material for: Segregation distortion: Utilizing simulated genotyping data to evaluate statistical methods
Source: PLoS One. 2020 Feb 19;15(2):e0228951. doi: 10.1371/journal.pone.0228951 (PMC7029859; doi:10.1371/journal.pone.0228951)

S3 Fig. Examining the effect of selection position on the number of simulations containing significantly distorted markers. Position 100 is in a region of low recombination, whereas position 200 is in a region of high recombination. Columns are separated by the type of statistical test performed, whereas rows are separated by population size. The FDR correction has consistently more power when selection is in position 100. sig. = significant, dist. = distorted, S. = selection, Pos. = position

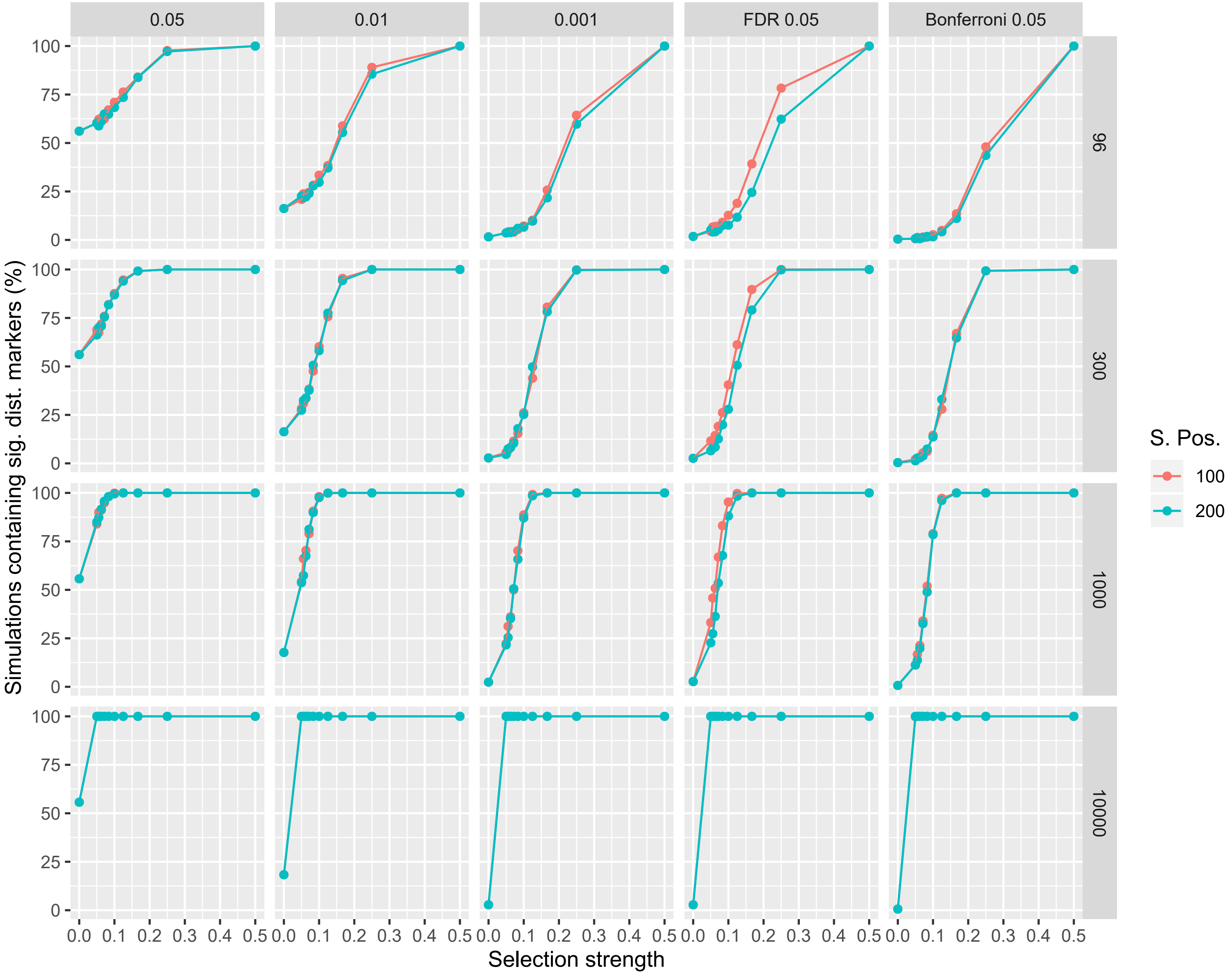

Supplement: S3 Fig — Position 100 is in a region of low recombination, whereas position 200 is in a region of high recombination. Columns are separated by the type of statistical test performed, whereas rows are separated by population size. The FDR correction has consistently more power when selection is in position 100. sig. = significant, dist. = distorted, S. = selection, Pos. = position. (PDF) [file pone.0228951.s003.pdf]
