# Supplementary material for: Segregation distortion: Utilizing simulated genotyping data to evaluate statistical methods
Source: PLoS One. 2020 Feb 19;15(2):e0228951. doi: 10.1371/journal.pone.0228951 (PMC7029859; doi:10.1371/journal.pone.0228951)

S4 Fig. Comparison of the effect of selection position on formation of segregation distortion regions (SDRs). Selection position 100 is in a region of low recombination, whereas position 200 is a region of high recombination. (a) shows the total number of SDR among 1000 simulations as a function of selection strength, whereas (b) shows the number of simulations with at least 1 SDR. Shown in the panel titles are the thresholds / type of statistical tests used to detect segregation distortion. num. = number, sim. = simulations

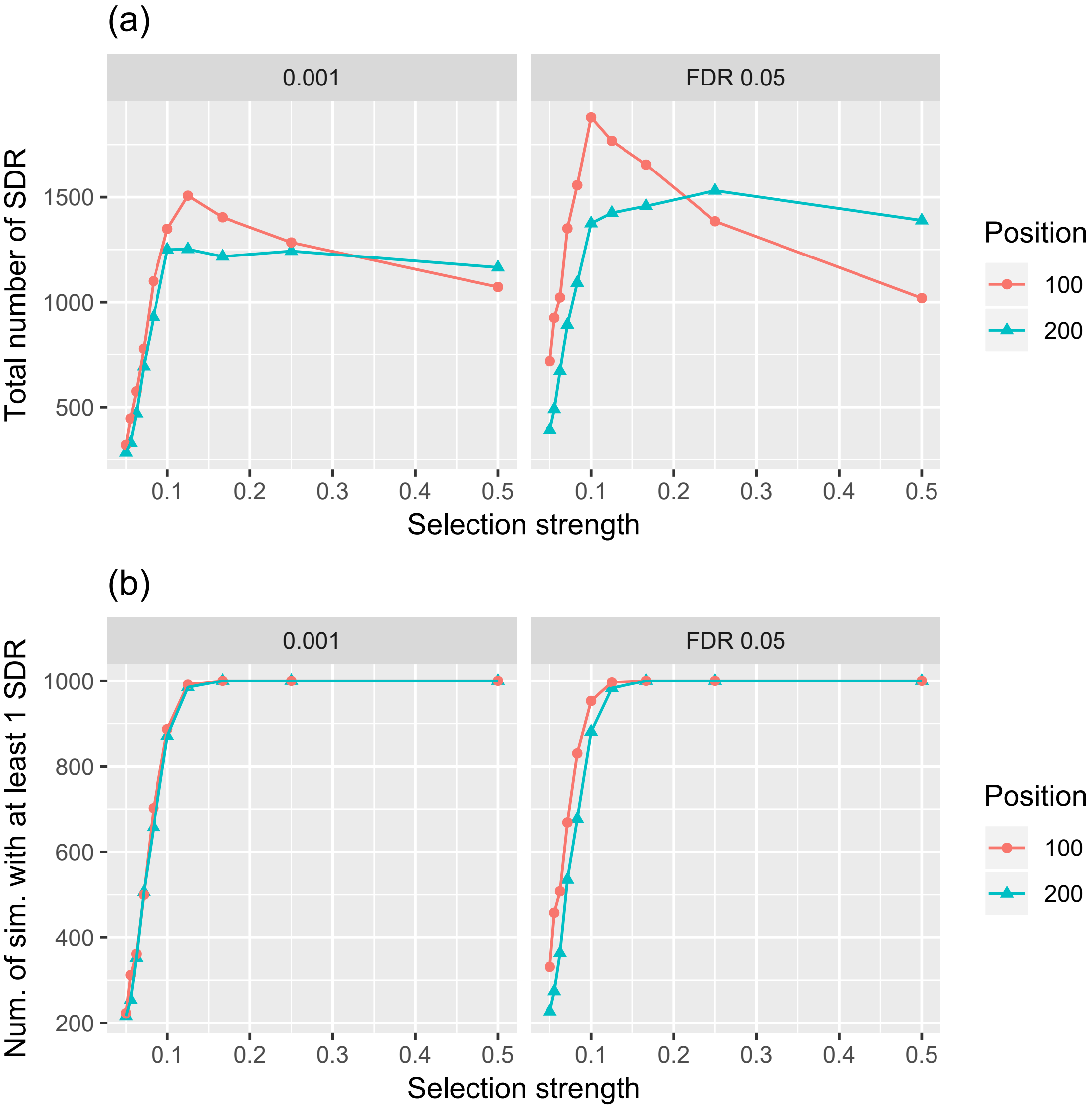

Supplement: S4 Fig — Selection position 100 is in a region of low recombination, whereas position 200 is a region of high recombination. (a) shows the total number of SDR among 1000 simulations as a function of selection strength, whereas (b) shows the number of simulations with at least 1 SDR. Shown in the panel titles are the thresholds / type of statistical tests used to detect segregation distortion. num. = number, sim. = simulations. (PDF) [file pone.0228951.s004.pdf]
